# Comparative analysis of acoustic therapies for tinnitus treatment based on auditory event-related potentials

**DOI:** 10.3389/fnins.2023.1059096

**Published:** 2023-04-04

**Authors:** Luz M. Alonso-Valerdi, David I. Ibarra-Zárate, Alma S. Torres-Torres, Daniela M. Zolezzi, Norberto E. Naal-Ruiz, Janet Argüello-García

**Affiliations:** ^1^Tecnológico de Monterrey, Escuela de Ingeniería y Ciencias, Monterrey, Mexico; ^2^Unidad Profesional Interdisciplinaria en Ingeniería y Tecnologías Avanzadas, Instituto Politécnico Nacional, Mexico City, Mexico

**Keywords:** tinnitus, acoustic therapy, auditory event related potential (AERPs), area under a curve (AUC), audition (test), hospital anxiety and depression scale (HADS), tinnitus handicapped inventory (THI)

## Abstract

**Introduction:**

So far, Auditory Event-Related Potential (AERP) features have been used to characterize neural activity of patients with tinnitus. However, these EEG patterns could be used to evaluate tinnitus evolution as well. The aim of the present study is to propose a methodology based on AERPs to evaluate the effectiveness of four acoustic therapies for tinnitus treatment.

**Methods:**

The acoustic therapies were: (1) Tinnitus Retraining Therapy (TRT), (2) Auditory Discrimination Therapy (ADT), (3) Therapy for Enriched Acoustic Environment (TEAE), and (4) Binaural Beats Therapy (BBT). In addition, relaxing music was included as a placebo for both: tinnitus sufferers and healthy individuals. To meet this aim, 103 participants were recruited, 53% were females and 47% were males. All the participants were treated for 8 weeks with one of these five sounds, which were moreover tuned in accordance with the acoustic features of their tinnitus (if applied) and hearing loss. They were electroencephalographically monitored before and after their acoustic therapy, and wherefrom AERPs were estimated. The sound effect of acoustic therapies was evaluated by examining the area under the curve of those AERPs. Two parameters were obtained: (1) amplitude and (2) topographical distribution.

**Results:**

The findings of the investigation showed that after an 8-week treatment, TRT and ADT, respectively achieved significant neurophysiological changes over somatosensory and occipital regions. On one hand, TRT increased the tinnitus perception. On the other hand, ADT redirected the tinnitus attention, what in turn diminished the tinnitus perception. Tinnitus handicapped inventory outcomes verified these neurophysiological findings, revealing that 31% of patients in each group reported that TRT increased tinnitus perception, but ADT diminished it.

**Discussion:**

Tinnitus has been identified as a multifactorial condition highly associated with hearing loss, age, sex, marital status, education, and even, employment. However, no conclusive evidence has been found yet. In this study, a significant (but low) correlation was found between tinnitus intensity and right ear hearing loss, left ear hearing loss, heart rate, area under the curve of AERPs, and acoustic therapy. This study raises the possibility to assign acoustic therapies by neurophysiological response of patient.

## 1. Introduction

Tinnitus is a complex perception unrelated to an external sound stimulus. Its origin unravels in the central nervous system (CNS) beyond the auditory pathway, and even, the auditory cortex (Langguth et al., 2013). In most cases, cochlear damage, and hearing loss act as a trigger of the tinnitus percept (Kaltenbach, 2011). However, this mechanism is not sufficient to explain its sustained presence in the CNS. Tinnitus has been explained by cortical reorganization following deafferentation (Weisz et al., 2007) that follows peripheral hearing loss in accordance with the Edge Theory (Han et al., 2009). Deafferentation is the first step toward the reorganization of the auditory cortex, which is further supported by the acoustic characteristics of the tinnitus percept that correspond to the region of hearing loss (Sereda et al., 2011). The second step would be any of these three mechanisms of plastic changes: (1) strengthening of existing synapses, (2) awakening of dormant synapses, or (3) growth of new connections (Adjamian et al., 2014). In previous studies (Jastreboff, 1990) and recent models (Vanneste et al., 2019) that explain the pathophysiology of tinnitus, it has shown that neurons that lack of an input start responding to inputs from nearby frequency regions, and thereby, replace their own characteristic frequency. In turn, neuronal synchronicity is increased because a disproportionately large population of neurons are responding to the same input (Eggermont and Roberts, 2004). This is the role of increased spontaneous activity (SA) that affects multiple layers of auditory brain network proposed by Noreña and Farley (2013). They suggest that subcortical structures amplify the ongoing SA in the peripheral system. If tinnitus becomes chronic, neural changes are independent of peripheral auditory structures, and more related to neuronal activity from central processing networks. For example, the subcortical structures are deeply intertwined to emotional processing networks which are related to the chronicity of tinnitus (Carpenter-Thompson et al., 2015).

### 1.1. SA changes in neural oscillations due to tinnitus

Some of SA changes in neuronal activity has been observed in electroencephalogram (EEG) monitoring. For example, *delta* (0–4 Hz) and *theta* (4–8 Hz) band oscillations were enhanced due to tinnitus loudness and tinnitus-related affliction. Patients suffering from a greater tinnitus affliction showed larger *theta* oscillations (Balkenhol et al., 2013). According to Milner et al. (2020), most tinnitus patients showed a decrease in *alpha* (8–13 Hz) power, possibly owing to the redirection of their attention toward their tinnitus during mind wandering. Synchronization of *beta* (13–30 Hz) band rhythms has been detected as indicative of chronic dysrhythmia of thalamus cortical circuits following auditory deafferentation, and tinnitus-related affliction (De Ridder et al., 2015; Milner et al., 2020). Most EEG studies relate chronic tinnitus with higher *gamma* band (>30 Hz) activity associated with loudness, attention or emotions generated by tinnitus awareness (Loo et al., 2009; Ridder et al., 2011; Sedley et al., 2012; Meyer et al., 2014; Milner et al., 2020).

### 1.2. Auditory event-related potentials (AERPs)

A wide variety of factors [e.g., blood glucose changes (An et al., 2015), menstrual cycle (Solís-Ortiz et al., 1994), and prolonged wakefulness (Hung et al., 2013)] influence modulation of electroencephalographic SA monitoring. Consequently, it is difficult to exclusively associated tinnitus alterations with observed EEG patterns. Therefore, experimental paradigms based on evoked and induced activity for EEG analysis are frequently preferred in order to relate neural responses with specific emotional, cognitive, motor, perceptual and sensory events.

A traditional way to measure evoked activity is based on event-related potentials (ERPs). These potentials emerge subsequently to the onset of a sensory, cognitive, or motor event, and are phase and time locked. Typically, ERPs are assessed by their amplitude, latency (early, medium, or late), topographical distribution, and sensitivity (Kropotov, 2010; Luck and Kappenman, 2011). The amplitude of these evoked responses depends on the action potentials of post-synaptic neural connections, and the number of neurons engaged in the processing of the stimulus. ERPs may be exogenous or endogenous. Exogenous components appear within 100 ms after stimulus onset, and they are defined by the physical characteristics of such stimulus (e.g., intensity, tone, frequency, pitch, and timbre). Endogenous components depend on psychological variables such as attention or task relevance and seem to have perceptual importance given their change by cognitive factors. In particular, auditory ERPs (AERPs) originate from primary cortical areas and depend on the cognitive state of the individual. Some typical AERP components are P1, N1, P2, and N2, which, respectively, occur 50, 100, 170, and 250 ms after auditory stimulus onset (Cardon et al., 2020).

Regarding tinnitus research based on AERPs, three long latency peaks have been crucial in the study of tinnitus brain function: N1, N2, and P3 (Rogers et al., 1991). A brief review regarding the findings of AERPs in tinnitus as compared to controls is summarized in Table 1. Amplitudes are reported in microvolts (μV), and latency are in milliseconds (ms). In the following values reported, some studies differ from others due to diversity in methodologies applied (e.g., paradigm, frequency, and volume of tones).

**TABLE 1:** Comparison of amplitudes and latencies of AERP components between controls and tinnitus patients.

References	Type of paradigm	Frequency, volume, and number of stimuli	Amplitude and latency of components compared to controls	
			**Tinnitus**	**Controls**
Attias et al., 1993	Repetitive	1 kHz of 200 stimuli at 75 dB	**N1**:2.3 μN **P2**:162 ms and 1.5 μV	**N1:**4.2 μV **P2:**182 ms and 2.5 μV
	Oddball paradigm	**Target stimuli:** 1 kHz **Non-target stimuli:** 2 kHz	**N1:**2.5 μV **P3:**2.1 μV	**N1:**3.9 μV **P3:**4.8 μV
			**No difference found in latency**	
		Modified oddball	**Target stimuli:** 1 kHz **Non-target stimuli:** 5, 6, and 7 kHz (with their hearing loss and tinnitus pitches)	For the target stimulus, all components were reduced for tinnitus group. **No difference in latency**	
Jacobson and McCaslin, 2003	Selective auditory attention	250 stimuli of 500 Hz and 1 kHz pure tones at 60 dB	**N1:**−2.20 μV and 111.79 ms	**N1:**−2.88 μV and 110.1 ms
	Ignore		**N1:**−1.73 μV and 108.07 ms	**N1:**−2.22 μV and 112.82 ms
	Passive listening paradigm		**N1:**−1.93 μV and 108.54 ms	**N1:**−2.48 μV and 109.54 m
Santos Filha and Matas, 2010	Acoustic stimulus tone burst	300 stimuli at 75 dB of 1 kHz (frequent) and 1.5 kHz (rare)	**N1** (RE): 142.8 ms **P2** (RE): 195 ms **P3** (RE): 326 ms	**N1** (RE): 90.8 ms **P2** (RE): 187 ms **P3** (RE): 316 ms
Said, 2012	Oddball paradigm	300 stimuli pure tone at 80 dB of 1 kHz (frequent) and 2 kHz (rare)	**P2**: 5.37 μV and 203.2 ms **P3**: 6.5 μV and 366.5	**P2**: 6.7 μV and 181.4 ms **P3**:10.6 μV and 320.6 ms
Elmorsy and Abdeltawwab, 2013	Oddball paradigm	Tone burst at 75 dB of 2 Khz (rare) and 1 kHz (frequent)	**P3**: 8 μV and 316 ms	**P3**: 9.4 μV and 306 ms
Houdayer et al., 2015	Oddball paradigm	330 tone burst stimuli binaurally presented of 1 kHz (frequent) and 2 kHz (rare)	**N1**: −7.77 μV and 104 ms **P2**: 6.43 μV and 185 ms	**N1**: −8.5 μV and 116 ms **P2**: 8.36 μV and 196 ms

**FIGURE 1:**
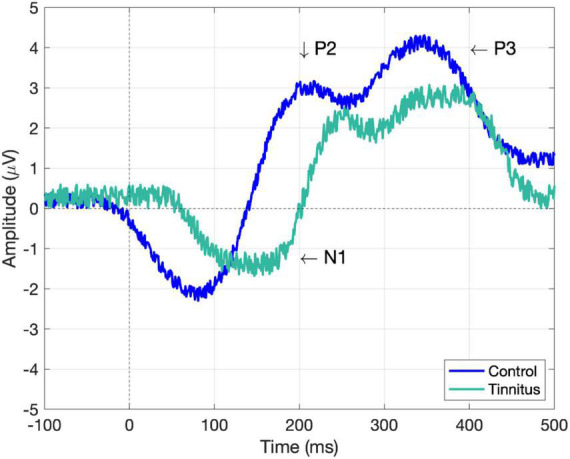
Simulated AERP components (N1, P2, and P3) of tinnitus and healthy controls. The approximated values according to the previous literature are the following: N1 for control (–2.2 μV, 80 ms) and tinnitus (–1.7 μV, 150 ms), P2 for control (2.9 μV, 207 ms) and tinnitus (2.4 μV, 250 ms), and P3 for control (4 μV, 340 ms) and tinnitus (2.6 μV, 380 ms). Note, amplitude and latencies could vary according to the applied methodology. Control is depicted in blue and tinnitus in cyan (greenish blue).

Clinically, component N1 has been used as an indicator of other neural deficits since the amplitude of the component in comparison with healthy individuals was found to be reduced in schizophrenia (Foxe et al., 2011), stroke (Ye et al., 2019), and epilepsy (Ford et al., 2001; Sun et al., 2008) patients. In addition, latency had been previously reported as an increased parameter in epilepsy patients (Drake et al., 1986). N1, P2, and P3 have been the most commonly used components to quantify the effects of tinnitus at a cortical level (Azevedo et al., 2020) since it has been demonstrated that their amplitudes are lower in tinnitus sufferers (Shiraishi et al., 1991; Attias et al., 1993; Elmorsy and Abdeltawwab, 2013). In a study using passive and selective auditory attention, N1 amplitudes for tinnitus were smaller in both conditions, while being higher only in the attentive condition for controls. As all auditory signals are transmitted through an upstream auditory pathway to be processed, the diminution in amplitude, and increase in latency may be a result of the upstream adaptation that occurs because of the continuous afferent signals (i.e., tinnitus) (Jacobson and McCaslin, 2003). P3 reflects higher order brain functions, fundamentally cognitive processes (Linden, 2005). In fact, the insula and upper cortical areas are the locations responsible for the generation of auditory P3 response (Azevedo et al., 2020). Auditory P3 peak amplitudes are reduced in patients with idiopathic tinnitus compared to normal subjects, but P3 peak latencies were not statistically significant (Elmorsy and Abdeltawwab, 2013). The lower amplitudes of N1 and P3 components are inferred to be a consequence of their redirected attention, which do not permit neurons to synchronize toward an attention stimulus, especially in high distress tinnitus (Delb et al., 2008). In fact, patients with tinnitus pay an increased attention to their interior sound, even when they are surrounded by noise, which is why tinnitus worsens into a silent environment. Similar findings regarding the higher latencies (N1, P2, P3) and amplitude reduction (P2, P3) have been observed in tinnitus with sensorineural hearing loss (Attias et al., 1993; Jacobson and McCaslin, 2003; Santos Filha and Matas, 2010; Said, 2012; Elmorsy and Abdeltawwab, 2013; Houdayer et al., 2015). Refer to Table 1. However, patients with sensorineural hearing loss without tinnitus presented no alteration, as healthy subjects (Said, 2012). Differences between bilateral tinnitus and unilateral tinnitus have also been described, with the latency of N1 being reduced in bilateral tinnitus, while the amplitude of N1–P2 increased in the ear with unilateral tinnitus (Santos Filha and Matas, 2010; Houdayer et al., 2015). Peripheral hearing loss can directly affect the P3 latency as well as the latency and amplitude of the waves N1 and N2 (Hall et al., 1990), but apparently P3 is not influenced by moderate peripheral hearing loss. Auditory brainstem responses (very small AERPs originating from the auditory nerve and brainstem) have been used to show abnormal activity in patients with tinnitus, being this abnormality expressed in specific features, such as prolonged inter-peak, and inter-aural latencies in wave V (Said, 2012), or smaller wave I amplitudes (Konadath and Manjula, 2016; Bramhall et al., 2019). In Figure 1, the most significant findings based on the components N1, P2, and P3 to evaluate the perceptual and cognitive state of tinnitus sufferers are represented.

### 1.3. AERPs as neuromarkers for monitoring tinnitus evolution

So far, AERP features have been used to characterize neural activity of patients with tinnitus. However, these EEG patterns could be used to evaluate tinnitus evolution as well. This is an interesting proposal to evaluate the effectiveness level of tinnitus treatments since neuroplastic changes (e.g., reduction in latencies of components, or an increase in their amplitudes) are feasibly detectable.

On this evidence, AERPs are herein proposed to evaluate neuroplastic changes owing to sound effects on tinnitus. Indeed, previous studies have been in line with this evaluation proposal. As a case in point (Poremski and Kostek, 2012), used auditory brainstem potentials for the evaluation of ultrasound in one patient who reported disappearance of tinnitus. AERPs have been also used to evaluate changes before and after 1 week treatment with repetitive transcranial magnetic stimulation (rTMS) in tinnitus patients (Yang et al., 2013). Pre-treatment tinnitus amplitude of N1 was larger in standard stimuli than in deviant stimuli. Similarly, tinnitus patients had smaller mismatch negativity (MMN), and late discriminative negativity (LDN) at Fz (EEG channel over the frontal lobe) compared to control. Topographic maps before rTMS demonstrated global asymmetry between the left and right cerebral hemispheres with more negative activities on left hemisphere, and more positive activities on right hemisphere. After rTMS treatment, neural activity of patients and controls were symmetrically comparable. In addition, these patients showed an increase of amplitude in P1, MMN, and LDN. After rTMS treatment, tinnitus patients showed increased N1 response to deviant stimuli, and larger MMN and LDN compared with pre-treatment (Yang et al., 2013).

As can be seen, AERPs are EEG patterns that could be used as objective indicators to monitor the effectiveness of tinnitus treatment, acoustic therapies for this study (Ibarra-Zarate and Alonso-Valerdi, 2020). The aim of the present study is, therefore, to propose a methodology based on AERPs to evaluate the effectiveness of four typical acoustic therapies for tinnitus treatment. The acoustic therapies were: (1) tinnitus retraining therapy (TRT), (2) auditory discrimination therapy (ADT), (3) therapy for enriched acoustic environment (TEAE), and (4) binaural beats therapy (BBT). Additionally, these four acoustic therapies are compared with the effect of relaxing music (a usual self-medicating sound-based treatment) in both tinnitus sufferers and healthy individuals. The investigation is described in detail below.

## 2. Materials and methods

The present research was undertaken as follows. Firstly, a methodology to implement personalized acoustic therapies (TRT, ADT, TEAE, and BBT), and to monitor their effect on the basis of AERP analysis was developed. The monitoring was supported by a questionnaire-based evaluation. The questionnaire-based evaluation relayed on the Tinnitus Handicapped Inventory (THI) and the Hospital Anxiety and Depression Scale (HADS). The thorough evaluation can be consulted in Alonso-Valerdi et al. (2021). Secondly, resulting AERPs before and after the acoustic therapy in use were estimated to evaluate the level of acoustic therapy effectiveness. Thirdly and finally, the acoustic therapies were ranked in accordance with their effectiveness level determined by the AERP properties (amplitude and topographical distribution).

### 2.1. Acoustic therapies

#### 2.1.1. Tinnitus retraining therapy (TRT)

Tinnitus retraining therapy is an acoustic therapy developed by Jastreboff that seeks to reduce tinnitus by reducing the loudness perception of the unreal sound. It intends to habituate the limbic system to reactions toward tinnitus (Jastreboff and Jastreboff, 2000; Kim et al., 2014). A random noise signal is used, and is additionally filtered by octave bands, depending on the tinnitus frequencies, and hearing loss in each ear. The noise signal is played at a level below the perceived level of tinnitus, producing a reduction in tinnitus loudness. The gradient between signals causes this psychoacoustic effect. When tinnitus is perceived with a background sound with a lower level, tinnitus perception level is reduced compared to tinnitus alone; and therefore, neural activity related to tinnitus alone is reduced (Jastreboff and Jastreboff, 2000). Improvements have been observed within 8 weeks (Kim et al., 2014), 6 months (Lee et al., 2019), 18 months (Lee et al., 2018), and up to 24 months after starting the treatment (Jastreboff and Jastreboff, 2000). Other improvements have been demonstrated when combining TRT with hearing aids (Bauer et al., 2017), and other colors of noise such as white, pink, and red (Barozzi et al., 2017).

#### 2.1.2. Auditory discrimination therapy (ADT)

This therapy intends to redirect the patient attention toward other sensorial events different from tinnitus to reduce its perception. It redirects the attention patient toward the therapy by presenting a composed sound of standard and deviant pulses in a random way. The patient must identify which type of pulse is presented, either standard or deviant. The standard pulse is the same tone that the tinnitus is, and the deviant pulse is 10% more than the standard one. Auditory discrimination has shown improvement in tinnitus symptoms attributed to rehabilitation of auditory processing frequencies of the auditory cortex damaged due to tinnitus (Herraiz et al., 2010), and prevention of auditory cortex reorganization (Flor et al., 2004).

#### 2.1.3. Therapy for enriched acoustic environment (TEAE)

Therapy for enriched acoustic environment is based on selective stimulation and intends to prevent hearing loss (according with audiometric curve) and subsequent plastic tonotopic cortical map changes after acoustic trauma. TEAE is based on a sequence of random frequency tones (burst and pip pulses) with amplitude proportional to the hearing loss reported on audiometry of patients. Frequency pulses stimulate the auditory pathway in a selective and personalized way. The stimulation is selective, because each tone of the sequence has a frequency-response curve very similar to the curves of neural resonances of the auditory pathway. It has been well documented that those tones contain three pure tones, which match the tinnitus frequency, and combined with white noise improve tinnitus symptoms (Tao et al., 2017). Similarly, fractal tones tailored to the hearing loss of the participants have demonstrated a decrease in tinnitus severity (Tyler et al., 2017), and prevention of tonotopic reorganization of the auditory cortex (Eggermont and Roberts, 2004; Noreña and Eggermont, 2005).

#### 2.1.4. Binaural beats therapy (BBT)

Binaural beats therapy is an acoustic treatment for tinnitus that is still being investigated by researchers. In the last decade, it has shown modulation of the neural activity for the reduction of stress (Munro and Searchfield, 2019). This therapy consists of two pure tones. One pure tone is presented on one ear, and the other one is presented on the opposite ear. The two tones have a difference in frequency according to the target oscillatory band. The use of this therapy has reported reduction of stress levels by reducing activation of areas in the sympathetic system (da Silva Junior et al., 2019), and induction of a relaxing state to palliate tinnitus side effects (David et al., 2010). Indeed, music therapy and BBT for tinnitus treatment were compared in Ibarra-Zarate et al. (2022). Authors concluded that BBT was a suitable acoustic therapy to reduce tinnitus distress in patients with no anxiety.

#### 2.1.5. Relaxing music

Music influences the structure and functions of the central nervous system and the neurovegetative system, endocrine glands and internal organs. The music effect mostly depends on several factors, including melody, harmony, rhythm, and timbre. Music as therapy has been mostly indicated for stress, socialization problems, physical, mental, and emotional disorders, and serves as a mood regulator (Benenzon, 2004). Based on Heidelberg model, music therapy may prevent tinnitus. For this investigation, relaxing music of 60−80 beats per minute for self-supporting tinnitus sufferers was selected.

### 2.2. Sample

Eighty nine patients with chronic and refractory tinnitus, and 14 healthy volunteers, were invited to participate in the clinical protocol described in Alonso-Valerdi et al. (2017), previously approved by the Ethical Committee of the Medicine School at Tecnologico de Monterrey (COFEPRIS13CI19039138) and registered as clinical trial in BioMed Central (ISRCTN14553550). The database is publicly available at https://data.mendeley.com/datasets/kj443jc4yc/1, and a further reference of the database can be found in Cuevas-Romero et al. (2022).

**FIGURE 2:**
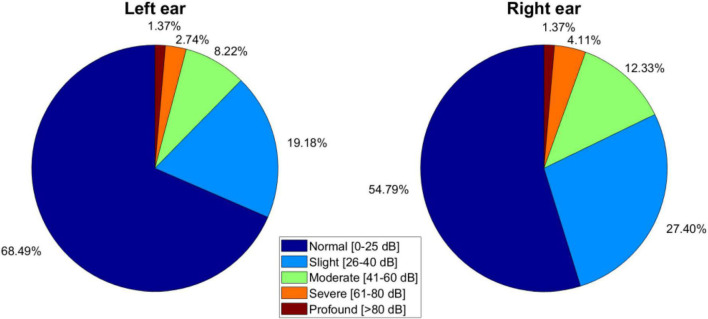
Hearing levels of the cohort of 71 tinnitus sufferers presented in five levels: (1) Normal hearing (0–25 dB), (2) slight (26–40 dB), (3) moderate (41–60 dB), (4) severe (61–80 dB), or (5) profound (> 80 dB) hearing loss.

**FIGURE 3:**
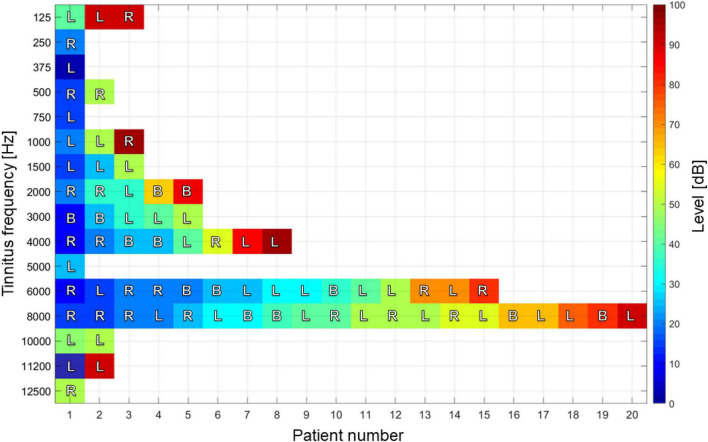
Tinnitus pitch, intensity, and recurrence of the cohort of 71 tinnitus sufferers. Decibel values indicate perceived sound level: from very soft (0) to very loud (100 dB). Letters show the laterality of tinnitus, left (L), right (R) or both (B) ears.

From the 103 patients, 53% were females and 47% were males. Of the cohort, 32 of them did not complete the procedure and were discarded from the study. In total, 71 participants were included in the present study (see Table 2). Age was not an exclusive criterion. Patients with normal audition, unilateral or bilateral hyperacusis, and/or conductive sensory-neural hyperacusis were included. In Figure 2, the audition level of the sample is reported. In addition to the audiometry studies, tinnitus identification was required. Pure tone test (audiogram) refers to identify frequency and intensity of tinnitus as follows: Patients are fitted with hearing aids connected to an audiometer. Pure tones at a specific frequency and volume are transmitted into each ear, one at a time. They are asked to indicate when they hear a sound. A graph of the minimum volume required to listen is made. A device called a bone oscillator is placed against the mastoid bone to assess bone conduction. The tinnitus test is performed in an acoustically isolated booth, and with the help of an audiometer, the audiologist emits different sounds so that the patients identify the sound frequencies (high or low) and the intensity (volume) that most resembles the tinnitus symptoms they perceive. In this test the patient also identifies the minimum volume of noise needed to “mask” the tinnitus. In Figure 3, intensity, frequency, and laterality of the tinnitus participants are presented. Clinical and demographic information of the cohort of 71 participants is outlined in Table 3.

**TABLE 2:** In total, 103 of participants accepted to participate in the study, and 71 of them completed the experimental procedure.

Group number	Acoustic therapy	Participant condition	Number of recruits	Number of recruits with completed Procedure
1	Placebo	Patient with tinnitus	16	11
2	BBT		18	14
3	TRT		18	15
4	EAE		18	15
5	ADT		19	8
6	Control	Healthy volunteer	14	8
TOTAL:			103	71

**TABLE 3:** Clinical and demographic information of the tinnitus sufferers recruited for this study.

Gender		Age (years)			Hearing loss [dB]				Tinnitus laterality		
**Men**	**Women**	**Young**	**Adult**	**Elderly**	**Normal Audition**	**Slight**	**Moderate**	**Severe**	**Left**	**Right**	**Both**
		Age <29	29 < age < 60	Age >60	0:20	20:40	40:60	>60			
34	39	3	44	26	9	15	5	5	17	11	6

### 2.3. Experimental procedure

#### 2.3.1. Recruitment and group assignment

All the participants were informed about the clinical protocol and signed a consent form before their participation. They were additionally notified that their head physician was also following up the investigation. Having been informed, patients were assigned to one of five groups: (1) TRT, (2) EAE, (3) ADT, (4) BBT, and (5) placebo. Patients with no severe hearing loss were randomly assigned to placebo and EAE groups. On the other hand, patients with a well-identified tinnitus pitch were included in TRT or ADT groups. Patients with tinnitus lower than 1 kHz were assigned to BBT. The control group (healthy volunteers) was asked to use the same relaxing music as placebo group.

#### 2.3.2. Acoustic therapy procedure

From previous research, it has been observed that the time frame of acoustic stimulation treatments presents a cumulative effect on patients; that is, by exposing participants to daily interventions, improvements are observed after follow-up sessions distributed in time. This information has been supported with monthly follow-up sessions (Jain and Jain, 2016; Lee et al., 2018). Nevertheless, studies including neural acquisition techniques have shown cortical responses in 1 day (Karino et al., 2006), 8 days (da Silva Junior et al., 2019), and 8 weeks (Rodríguez-León et al., 2022) of daily acoustic stimulation. Neuroplasticity with auditory stimulation has been observed after a month of daily exposure (Rodrigues et al., 2010; Grau-Sánchez et al., 2013; Sihvonen et al., 2017). For these reasons, improvements caused by therapies reflected on the cortical activity of tinnitus sufferers can be monitored in shorter terms by neural acquisition techniques when daily stimulation is applied. Although the effects of acoustic therapies are cumulative 30 min every day for 16 weeks or 60 min for 8 weeks, there are needed 60 days to make a habit (Arlinghaus and Johnston, 2018).

#### 2.3.2 Acoustic therapy adjustment

To design acoustic therapies, clinical history of patients was used as follows. First, BBT was assigned to all those patients with a tinnitus frequency lower than 1 kHz. This therapy was applied with a carrier signal at the tinnitus frequency of each patient. Second, the main elements that were considered to design TRT were three: hearing loss (Figure 3), tinnitus intensity, and pitch (Figure 2). A broadband noise was generated, and a passband filter was designed according to the frequency band and intensity of tinnitus. Third, TEAE was adjusted according to the hearing loss in full range of each patient, avoiding tinnitus pitch. Finally, ADT was adjusted according to tinnitus frequency for standard pulse and ±10% for deviant pulse, considering the hearing loss in such frequencies.

Patients were instructed to apply their assigned acoustic therapy for 1 h every day, at any time of the day, and for 8 weeks. Audio players were provided to patients for therapy purposes. Those audio players included a pair of headphones and a battery charger. Frequency response of the headphones was previously tested to guarantee sound quality. In addition, the volume limit was established in the mobile device according to the audiometry curve of each patient.

#### 2.3.3. Questionnaire-based monitoring

Acoustic therapies were monitored twice during the 8 weeks: before and after the treatment. At each monitoring session, the THI and HADS questionnaires were applied, and the results of the questionnaire-based evaluation can be found in Alonso-Valerdi et al. (2021). To support the AERP findings, the THI and HADS overall outcome is included in this study. For the first questionnaire, tinnitus perception was evaluated in five categories: (1) light, (2) mild, (3) moderate, (4) severe, and (5) catastrophic. For the second questionnaire, anxiety, and stress due to tinnitus was assessed in three categories: (1) normal, (2) borderline abnormal, and (3) abnormal.

#### 2.3.4. EEG monitoring

**FIGURE 4:**
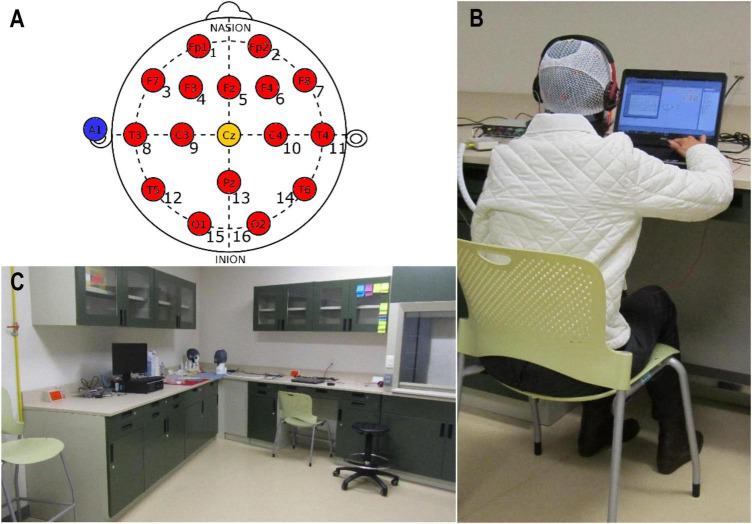
EEG acquisition: **(A)** EEG layout of 16 channels according to the 10/20 System (red circles), along with reference A1 (blue circle) and ground Cz (yellow circle); **(B)** g.USBamp amplifier of EEG signals; and **(C)** laboratory where the experimental procedure was undertaken. This laboratory was chosen because of the low background noise, which was moreover measured before testing and was around 35 dBA. This parameter is good enough to listen the acoustic therapies and to record EEG data.

**FIGURE 5:**
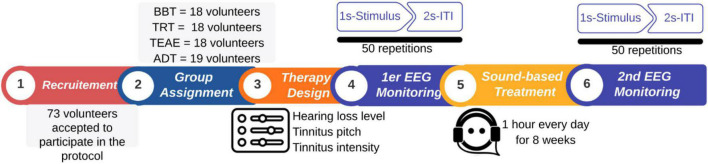
Organization of the experimental procedure. In total, 71 patients voluntarily participated in the present study. Patients followed up an 8 week sound-based program. An EEG monitoring was undertaken before and after the program to evaluate the effectiveness of acoustic therapy in use.

Acoustic therapies were monitored in the laboratory of Tecnológico de Monterrey (Figure 4C) twice during the 8 weeks: before and after the treatment. At each monitoring session, an EEG recording was undertaken. The procedure is depicted in Figure 5. For the EEG recording, participants were stimulated around 2 min with a sequence of 50 auditory stimuli of 1 s with an inter-trial interval (ITI) of 2 s. Participants were asked to keep their eyes closed during the auditory stimulation. Every auditory stimulus was in line with the acoustic therapy in use. It is important to note that EEG recordings at rest with eyes closed were additional taken before starting the sound-based treatment to estimate the individual alpha frequency (IAF) of each patient (see Figure 4B).

### 2.4. EEG signals: Acquisition and analysis

#### 2.4.1. Acquisition

To acquire the EEG signals, a g.USBamp EEG amplifier from g.tec Company (Graz, Austria) was used. Signals were recorded at 256 Hz in a bandwidth between 0.01 and 100 Hz. Sixteen EEG channels were collocated over the participant scalp in line with the 10/20 International System as illustrated in Figure 4A. The g.USBamp was connected with the computer via OpenViBE, an open-source software for real-time Neuroscience projects (Renard et al., 2010).

#### 2.4.2. Analysis

**FIGURE 6:**
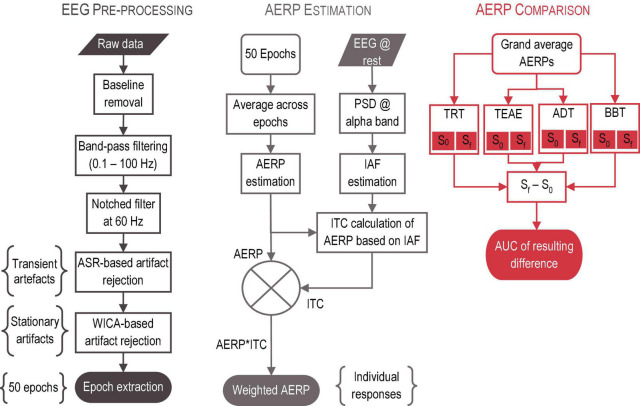
EEG signal analysis. The analysis was performed in three stages: (1) Pre-processing of EEG signals, (2) AERP estimation and validation based on ITC, and (3) AERP grand average per acoustic therapy and session.

To analyze EEG signals, EEGLAB toolbox for Matlab was used (Brunner et al., 2013), in addition to previously certified plug-ins, including Artefact Subspace Reconstruction (ASR) (Plechawska-Wojcik et al., 2018) and FastICA (Langlois et al., 2010). The analysis was performed in four stages: (1) Pre-processing of EEG signals, (2) AERP estimation based on inter-trial coherence (ITC), (3) AERP validation based on IAF, and (4) AERP grand average per acoustic therapy (BBT, TRT, TEAE, and ADT), and for both initial (S_0_) and final (S_f_) sessions. The analysis pathway is presented in Figure 6, and it is described in detail below.

*Stage 1: Pre-processing*. The main goal of signal pre-processing is to increase the EEG signal to noise ratio by eliminating external (e.g., line noise, patient motion) and internal (e.g., cardiac, and muscular electrical activity) artifacts. For this purpose, six steps were followed:

(1) baseline removal;
(2) band-pass filtering with an eighth order IIR Butterworth filter, and with a bandwidth of 99.9 Hz (0.1−100 Hz);
(3) 60 Hz component rejection with a second order IIR Butterworth filter;
(4) automatic rejection of transient artifacts (including, muscular activity, patient motion, and electrode pop-ups) by ASR algorithm;
(5) automatic elimination of stationary artifacts (including, ocular and cardiac activity) by the hybrid algorithm based on Wavelet analysis and Independent Component Analysis and proposed by Castellanos and Makarov (2006); and
(6) segmentation of EEG signals into 1 s time-windows, and in line with the 50 auditory stimuli described in Figure 2. Each resulting epoch was taken 200 ms before, and 800 ms after the stimulus onset.

*Stage 2: AERP Estimation*. Having extracted 50 time-windows for each patient, all of them were averaged to obtain their AERPs. Around IAF, ITC was calculated. ITC refers to the estimation of the level of repetitiveness of the transient and automatic response due to the auditory event. Ideally, such response must be found at every time-window, but in practice, repetitiveness at each trial depends on the stimulus characteristics and the experimental paradigm in use. The higher ITC, a more remarkable AERP is found. On this basis, AERPs were weighted by their resulting ITC to differentiate between a real component and a random peak.

*Stage 3: AERP Validation*. Three-min EEG recordings at rest of each patient were filtered between 7 and 14 Hz to estimate the power spectral density (PSD) over occipital lobe (O1 and O2). After estimating PSD from O1 and O2, both were averaged, and the peak value was detected to obtain the fundamental frequency of individual alpha band oscillation, that is, IAF. Note that this parameter (IAF) is a neural print, which is highly inheritable, and related to cognitive functioning (Grandy et al., 2013). It increases toward adultness and decreases toward elderly. It is larger in women than in men (Chiang et al., 2011). Weighting AERPs in accordance with the IAF compensate the demographic factors (e.g., age, and gender) that could be conditioning electrical features of AERPs.

*Stage 4: AERP Comparison*. AERPs were averaged across patients to obtain the grand average per therapy. Then, grand averages of S_f_ and S_0_ were subtracted each other, and the area under the curve (AUC) of the resulting difference was calculated.

### 2.5. Statistical evaluation

To evaluate whether the effect of acoustic therapies was significant, a statistical comparison before (S_0_) and after (S_f_) the therapy was undertaken as follows. Firstly, the normality of AERPs was tested by the D’Agostino-Pearson test (d’Agostino, 1971; d’Agostino and Pearson, 1973). Note that AUC estimated from grand average of AERPs for initial (S_0_) and final (S_f_) sessions per each of 16 EEG channels refers to the inputs of normality test. As was expected, input estimations were non-normal, and then, the Wilcoxon Rank Sum to test the null hypothesis (S_0_ = S_f_) was applied, considering two populations with 16 paired samples (i.e., 16 EEG channels). The null hypothesis was accepted if the significance level was equal to or more than 5% (*p* ≥ 0.05). A pseudocode of the statistical evaluation is presented in Table 4.

**TABLE 4:** Pseudocode of the statistical evaluation of acoustic therapy effect.

Statistical evaluation to test H_0_: S_0_ = S_f_
Initialize
Inputs
AUC (S_0_, 16 EEG channels) AUC (S_f_, 16 EEG channels)
Output
S_0_ = S_f_
S_0_ ≠ S_f_
Start
Normality testing based on D’Agostino-Pearson test
If no normality,* then
Statistical comparison between S_0_ and S_f_ based on Wilcoxon Rank Sum method
If *p* ≤ 0.05 then
S_0_ = S_f_
else
S_0_ ≠ S_f_
End

*No normality refers to no Gaussian distribution and non-parametric methods are necessary.

## 3. Results

To evaluate the effectiveness of acoustic therapies for tinnitus treatment, AERPs resulting from auditory stimulation based on the acoustic properties of the therapy were proposed in this work. AERPs reflect the level of neural synchronicity evoked by the acoustic therapy in use. Furthermore, it is important to bear in mind that patients with tinnitus have abnormal neural over-synchronicity owing to their condition. On this basis, an expected outcome is to observe larger AERPs before than after the treatment, if acoustic therapy was effective.

As AERPs are associated with the physical and temporal aspects of the stimulus, different acoustic therapies evoked different AERPs. To compare different AERP components, we then proposed to quantify and qualify the AUC by measuring magnitude and topographical distribution. In this way, the impact and localization of acoustic therapy effect was estimated and compared among different therapies.

Finally, the significance of the AUC differences between initial (S_0_) and final (S_f_) sessions to verify the acoustic therapy effect was measured by a non-parametric method (Wilcoxon Rank Sum) at a *p*-value less than 0.05. The resulting statistics are presented in Table 5. As can be seen from the table, TRT, ADT, and control groups showed a significant difference between the AERPs before and after the acoustic therapy.

**TABLE 5:** Statistical comparison between initial session (S_0_) and final session (S_f_) of resulting AUCs of grand average AERPs per acoustic therapy.

	Placebo	TRT	ADT	TEAE	BBT	Control
H_0_	S_0_ = S_f_	S_0_ = S_f_	S_0_ = S_f_	S_0_ = S_f_	S_0_ = S_f_	S_0_ = S_f_
*p*-values	0.576	0.001*	0.028*	0.216	0.133	0.037*

*TRT, ADT, and control groups showed significant difference before and after the treatment.

Additional to the electrophysiological reaction to acoustic therapies, the psychological response to those therapies has been included in this evaluation. For this purpose, tinnitus perception (THI outcome), and stress (HADS-S) and anxiety (HADS-A) owing to tinnitus were monitored before and after the acoustic therapy. The results are hereunder presented.

### 3.1. HADS and THI outcomes

**FIGURE 7:**
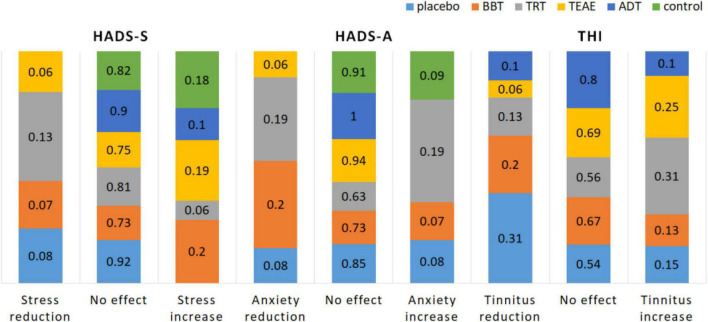
Psychological evaluation of acoustic therapies based on the monitoring of three factors: stress (HADS-S), anxiety (HADS-A), and tinnitus perception (THI).

Questionnaire-based monitoring of acoustic therapies are presented in Figure 7. According to the obtained outcomes before and after the treatment, a difference between the first session (S_0_) and the last one (S_f_) was estimated and categorized in: (1) positive effect that refers to the reduction of stress, anxiety, or tinnitus perception; (2) no effect; and (3) negative effect that refers to the increase of stress, anxiety, or tinnitus perception. A series of three bars per questionnaire (HADS-S, HADS-A, and THI) is presented in the figure. In most cases, no effect is shown. Stress, anxiety, and tinnitus perception did not change after the acoustic therapy in at least 73, 63, and 54% of patients, respectively. On the other hand, stress and anxiety yielded the major reduction in TRT group: 13% of patients and 19% of them, respectively. Tinnitus perception was mostly reduced by ADT with 31% of its population. Finally, stress increased in 20% of patients in control, TEAE, and BBT groups. Similarly, anxiety and tinnitus perception, respectively, increased in 20 and 31% of patients in TRT group.

### 3.2. AERP-based evaluation

#### 3.2.1. Placebo group

**FIGURE 8:**
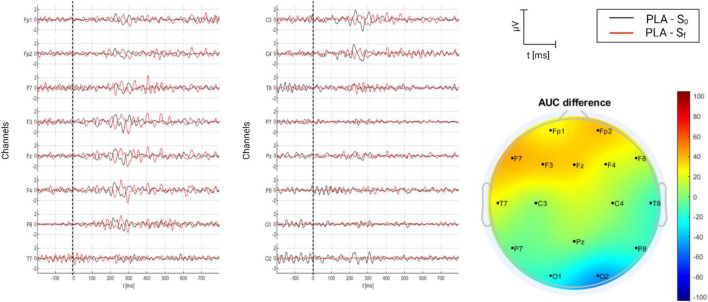
Grand average of AERPs at S_0_ (initial session) and S_f_ (final session) for placebo group (tinnitus sufferers treated with relaxing music). In addition, the AUC difference of these two grand averages (S_f_ – S_0_) is topographically plotted in μV.

Placebo group refers to tinnitus sufferers who were treated with relaxing music and no previous acoustical tuning. Therefore, music was a placebo. The grand average of AERPs for the placebo group is shown in Figure 8, where they are compared on time (S_0_ and S_f_), and the AUC difference per channel is depicted as well. From the figure, a P2–N2–P3 complex is present over the frontocentral region. The neural activity is much more synchronic in S_0_ than in S_f_ (AERPs are clearer), but the topographical distribution shows that a major difference over the frontal region in S_f_ was found.

#### 3.2.2. TRT group

**FIGURE 9:**
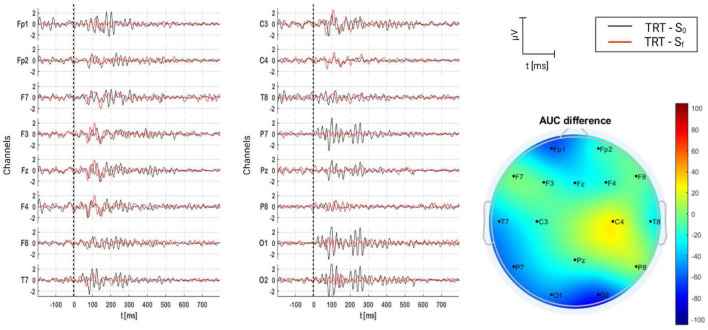
Grand average of AERPs at S_0_ (initial session) and S_f_ (final session) for TRT group. In addition, the AUC difference of these two grand averages (S_f_ – S_0_) is topographically plotted in μV.

The grand average of AERPs for the TRT group is shown in Figure 9, where they are compared on time (S_0_ and S_f_), and the AUC difference per channel is depicted as well. From the figure, several components are present in S_0_, particularly at Fp1, T7, P7, O1, and O2. Two complexes are identified: P1–N1–P2 and N2–P3–N3. In comparison, it seems that the level synchronicity diminished in S_f_, resulting in only one complex, P1–N1 present over the fronto-central region, particularly on C4. AUC differences reveal that AERPs were larger in S_f_ over the right somatosensorial region (C4), and they were smaller in S_f_ over the rest of recording sites. Indeed, these differences were significantly different (*p* = 0.001) (see Table 5).

#### 3.2.3. TEAE group

**FIGURE 10:**
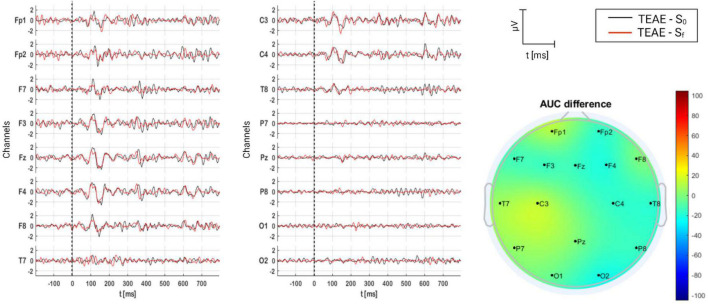
Grand average of AERPs at S_0_ (initial session) and S_f_ (final session) for TEAE group. In addition, the AUC difference of these two grand averages (S_f_ – S_0_) is topographically plotted in μV.

For TEAE group, the grand average of AERPs is shown in Figure 10, where they are compared on time (S_0_ and S_f_), and AUC per channel is topographically illustrated. Contrary to TRT group, P1 and N1 are clear components in both sessions over the fronto-central region. However, no significant difference exists between S_f_ and S_0_ as can be expected from the topoplot (*p* = 0.216) (see Table 5).

#### 3.2.4. ADT group

**FIGURE 11:**
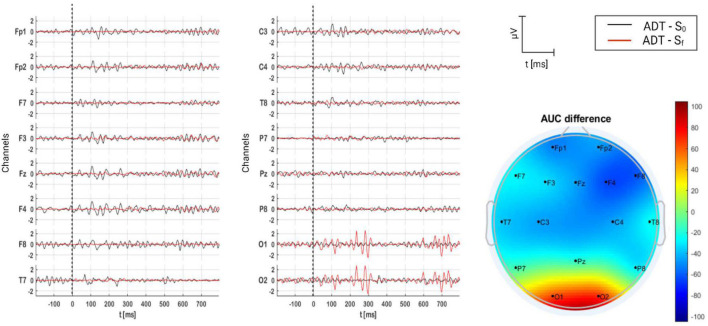
Grand average of AERPs at S_0_ (initial session) and S_f_ (final session) for ADT group. In addition, the AUC difference of these two grand averages (S_f_ – S_0_) is topographically plotted in μV.

The grand average of AERPs for ADT group is shown in Figure 11, where they are compared on time (S_0_ and S_f_), and AUC per channel is presented topographically as well. In terms of components, P1 is clear over the frontal region in S_0_, and N1, P2, N2, P3, and N3 are clear over the occipital region in S_f_. Statistically, AERP activity in S_f_ and S_0_ was different. AERPs were much larger in S_f_ over the occipital region.

#### 3.2.5. BBT group

**FIGURE 12:**
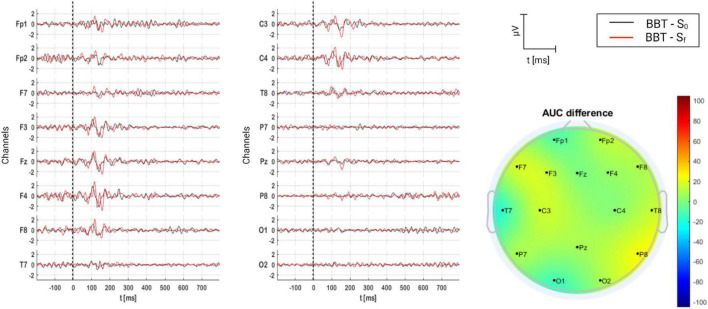
Grand average of AERPs at S_0_ (initial session) and S_f_ (final session) for BBT group. In addition, the AUC difference of these two grand averages (S_f_ – S_0_) is topographically plotted in μV.

Finally, for BBT group, the grand average of AERPs is shown in Figure 12, where they are compared on time (S_0_ and S_f_), and AUC per channel is presented as well. For this acoustic therapy, AERPs were composed of P1 and N1, appearing over the fronto-central region. Contrary to ADT group, no significant difference was found between sessions (*p* = 0.133) (see Table 5).

#### 3.2.6. Control group

**FIGURE 13:**
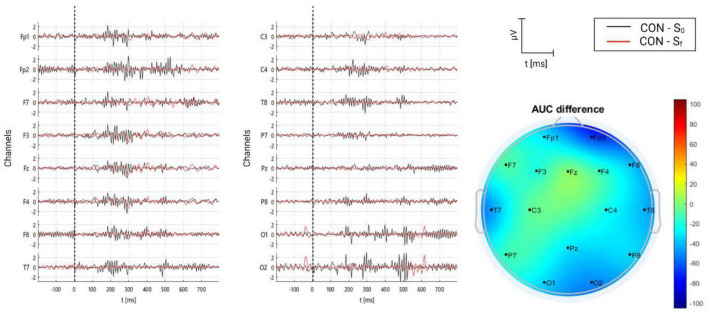
Grand average of AERPs at S_0_ (initial session) and S_f_ (final session) for control group (healthy individuals using relaxing music). In addition, the AUC difference of these two grand averages (S_f_ – S_0_) is topographically plotted in μV.

Similar to placebo group, participants in this group were treated with relaxing music. However, volunteers were individuals with no tinnitus who followed the experimental procedure, as did the rest of patients. In Figure 13, resulting AERPs on control group are shown. As can be seen, no clear complexes are present, as in the rest of group, neither in S_0_ nor S_f_. Regardless of no concreteness, the major neural activity was found in S_0_.

### 3.3. Correlation among demographic and audiological factors, AERPs, and acoustic therapies

**FIGURE 14:**
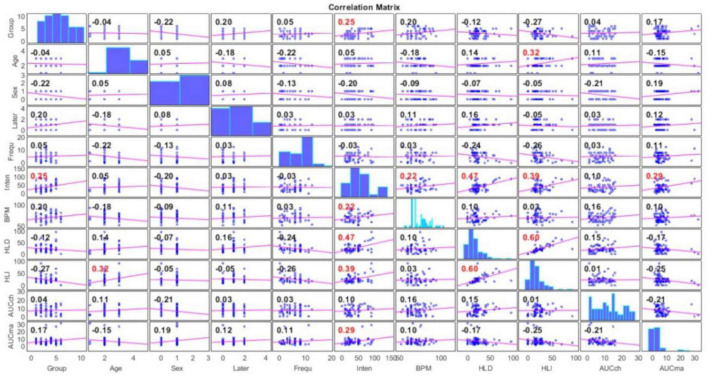
Correlation between demographic (age, sex) and audiological [tinnitus laterality, tinnitus frequency, tinnitus intensity, and hearing loss (both left and right)] information with magnitude (AUCmax) and topographical distribution (AUCch) of AERPs. Significant correlations are in red color.

Demographic (age, sex) and audiological [tinnitus laterality, tinnitus frequency, tinnitus intensity, and hearing loss (both left and right)] information was correlated with magnitude (AUCmax) and topographical distribution (AUCch) of AERP attribute, that is, AUC. The results are shown in Figure 14. As can be seen from the figure, the resulting correlated variables were:

• tinnitus intensity and acoustic therapy (ρ = 0.25);
• tinnitus intensity and heart rate in beats per minute (ρ = 0.22);
• tinnitus intensity and hearing loss on right ear (ρ = 0.47);
• tinnitus intensity and hearing loss on left ear (ρ = 0.39);
• tinnitus intensity and maximum AUC difference (ρ = 0.29);
• hearing loss on left ear and age (ρ = 0.32).

In the figure, significant correlations are shown in red font. As can be seen, the highest correlation was yielded between tinnitus intensity and hearing loss on right ear.

## 4. Discussion

### 4.1. How to evoke a brain response related to the sound effect of acoustic therapies?

Currently, there are very few researchers concerning the effectiveness validation of tinnitus treatments with AERPs. AERPs were used mainly to find abnormalities in amplitudes and latencies between controls and tinnitus patients, but not to evaluate treatment effects. In all those studies, the most common method for evoking potentials was the oddball paradigm, and the most common stimuli in use was a 1 kHz pure tone, either as the target stimulus (Attias et al., 1993) or as a constant sound (Santos Filha and Matas, 2010; Said, 2012; Yang et al., 2013). There are many reasons to choose this sound as the experimental stimulus. For example, it is fundamental for the calibration of audio devices. Moreover, it is the reference for the perception of loudness. This can be observed in the equal-loudness contours developed by Fletcher and Munson (1933), where the reference is at 1 kHz for loudness sensation. Furthermore, there is a wide range between the threshold of quiet and the threshold of pain for this pure tone. Finally, sensitivity at this frequency has lower probabilities to bring about hearing loss than frequencies above 3 kHz (Fastl and Zwicker, 2007).

However, the AERP experimental paradigm could not be suitable to evaluate the acoustic therapy effect since this standard evoked response is not reflecting how patients are processing the acoustic therapy in use. As a case in point, a recent study showed that this traditional AERP method cannot be considered as criterion to evaluate the evolution of drug treatment in patients with tinnitus (de Azevedo et al., 2021). Possibly, auditory stimulus similar to the tinnitus sound could be the ideal one to observe amelioration or aggravation on the tinnitus perception. However, the individual tinnitus is still known, acoustically speaking. That is, intensity and frequency composing of tinnitus is only known by the patient, but these acoustics parameters cannot be measured directly up to now.

In the light of the above discussion, we propose to evoke a brain response related to the sound-based treatment effect by stimulating based on the acoustic therapy in use. If acoustic therapies were being effective, resulting AERPs should have been different before and after the 8 month treatment since therapies pursue to

• reduce tinnitus perception (TRT) or
• rehabilitate hearing level (TEAE) or
• reduce tinnitus attention (ADT) or
• diminish tinnitus distress (BBT).

### 4.2. What to measure in AERPs to compare sound effects of different acoustic therapies?

Having decided to evoke AERPs based on the acoustic therapy in use, the next question to answer was: how to compare different AERPs resulting from different acoustic therapies? It is well established that AERPs reflect the auditory decoding at stimulus frequency and are shaped according to the acoustic features of sounds. For example, the sound given to the participants in TRT is wideband noise filtered in one-octave band centered at the tinnitus characteristic frequency. These signals appear to have more components in the shape of “noise” compared to the brain responses in BBT, but these extra components result from a more complex sound. The stimulus in BBT were two pure tones, one on each ear, which differed in frequency. Compared to the filtered noise, the amount of auditory information in this sound is much less since only two sounds are given.

Therefore, we proposed to quantify and qualify the AUC by measuring amplitude and topographical distribution to compare different AERP components. AUC reflects the magnitude of the level of neural synchrony related to an event, regardless of the morphology and latency of the potential, what makes possible to compare among different AERPs resulting from different acoustic therapies. In addition, the recording site of each AUC provides the topographical distribution of the neural information.

### 4.3. Which acoustic therapy was more effective (electroencephalographically speaking)?

As stated before, the cause of tinnitus is sustained far beyond the auditory system and encompasses physiological and emotional factors besides the atypical cognitive processing of auditory stimuli. Therefore, tinnitus patients are more prone to dysfunctional recognition, analysis, and cognitive processing of external and internal auditory stimuli (Haider et al., 2018). The presence of adaptive brain processes that occur for tinnitus patients, generated subcortically results in upstream adaptation as other auditory signals are processed. It has been hypothesized that the generators of AERPs are in a relative refractory state in which they are unable to respond fully to a transient auditory stimulus (Sereda et al., 2011). AERP tendencies of each acoustic therapy are discussed below.

#### 4.3.1. Relaxing music

Relaxing music was applied to tinnitus sufferers (placebo group) and participants with no tinnitus (control group). In this study, relaxing music was not tuned to acoustic parameters of tinnitus, and then, it is considered as a placebo sound. According to Simoes et al. (2019), the most applied therapy for tinnitus sufferers is the self-sound stimulation. They arbitrarily selected sounds (e.g., music, soundscapes) to mask their tinnitus without considering the side effects. The results of this study indicate that both groups (placebo and control) show a slight increase of synchronic neural activity over the left prefrontal region after the sound habituation. This slight change was only significant in the control group, as can be seen in Table 5. This result agrees with previous findings, suggesting that most auditory information integrates into the prefrontal cortex (Plakke and Romanski, 2014). On the other hand, 18 and 9% of control volunteers reported stress and anxiety increase after the sound habituation, respectively. Although 31% of tinnitus sufferers in placebo group reported reduction of tinnitus perception, this could be a placebo effect. Eventually, this sound stimulation could increase anxiety and stress, as in the control group. The significant AUC increase due to the sound habituation in the control group presented in this study supports this suggestion.

#### 4.3.2. TRT

The difficulty of habituating to the perceived sound causes major suffering for tinnitus patients. One study divided tinnitus patients in tinnitus complainers and non-complainers (Walpurger et al., 2003). They found that tinnitus complainers had ERP with higher amplitudes as opposed to non-complainers, meaning that patients with severe tinnitus fail to properly habituate to auditory stimuli following Hallam’s theory of habituation, and have an enhanced response in the amplitude of ERP components. As can be seen in Figure 9, and in Table 5, TRT significantly increased the AERP magnitude over the somatosensory region of the right hemisphere (C4). Thus, this result can be interpreted as an increase of synchronic neural activity, and in turn, a major tinnitus presence. The result is supported by 31% of patients who reported that their tinnitus perception increased after the TRT-based treatment (Figure 7). Furthermore, 20% of them reported anxiety increase, as well. Pérez-Bellido et al. (2018) demonstrated that auditory frequency representations can be distributed over brain regions traditionally considered to be dedicated to somatosensation, as in this study.

#### 4.3.3 ADT

Similar to TRT neuroimaging results, ADT showed significant differences before and after the therapy over the occipital region (Figure 11). AERP magnitude increased after the therapy. This is highly associated with the sound effect of ADT. As was described above, this therapy intends to redirect the patient attention toward other sensorial events different from tinnitus to reduce its perception. Rodríguez-León et al. (2022) demonstrated that attention toward tinnitus in tinnitus patients treated with ADT were significantly reduced after an 8 week treatment. Patients with low tinnitus intensity and low hearing loss presented the highest betterment. Therefore, it is not surprising to observe a greater AERP after the ADT, what clearly reflects that attentional resources are captured by therapy, instead of tinnitus. What is surprising is that this phenome is observable over the occipital region. This could be explained by the work of Shomstein (2004), who showed that attentional control functions of posterior parietal and superior prefrontal cortices are not limited to the visual domain, but they also include the control of shifts of attention based on vision and audition. This work stated that attention shifted by audition (ADT goal) can be reflected over the parieto-occipital region, as in the present investigation. Finally, this interpretation is supported by THI outcomes, where 31% of patients in ADT group reported that their tinnitus perception decreased. Furthermore, ADT volunteers were who fewer side effects reported (Figure 7), in comparison with the rest of groups.

#### 4.3.4. TEAE

In contrast to other therapies, the auditory P300 presented subcomponents in TEAE. P300 appears when the patient is unexpectedly presented with a target stimulus during a stimulus discrimination task (Polich, 2007). P300 is now understood as a unified phenomenon that is composed of several parts that reflect an information processing cascade of attentional and memory mechanisms. After initial sensory processing, an attention comparison process evaluates the input with previous sensory experience in working memory. If a new stimulus is detected, then attentional processes activate to update the stimulus representation reflected in P300. This is also similar to the habituation/dishabituation process. Orienting response captures attention and rapidly habituates as events become familiarized (Yamaguchi et al., 2004). Novel stimuli detection involves the activation of a distributed neural network, involving association cortex and limbic system. This is because when a three-stimulus task with a distracter stimulus occurs infrequently, the subject is instructed to respond only to the target and otherwise refrain from responding. The distractor elicits a P3a and target elicits a P3b. However, AERP resulting before and after TEAE are not significantly different, as can be seen from Figure 10 and Table 5. These results suggest that the attentional resources used to process auditory TEAE information did not change, and possibly no auditory rehabilitation was achieved.

#### 4.3.5. TBB

The brain response to TBB seems to reflect the central mechanisms of auditory perception such as elementary and complex auditory perception. This response arises probably as early as the brainstem, in the superior olivary cortex, which is the first nucleus in the auditory ascending pathway to receive a bilateral input (Spitzer and Semple, 1998). TBB is also related to the brain’s spatiality to localize sound sources and track moving sounds from the information feedforwarded to the inferior colliculus (Gerken et al., 1975). TBB processing passes as a neuroelectric discharge that relays to the thalamus and the primary auditory cortex. It further involves secondary and non-specific auditory areas (amygdala and limbic system), which is why it has been proposed as an induction technique to habituate to tinnitus (David et al., 2010). In this study, there was an increased latency between the first and the final sessions for all components and leads, except for the right temporal lead where latency was decreased. Multiple source estimates from neuromagnetic responses were localized in the parietal, frontal and bilateral temporal cortices to binaural beat stimulation (Karino, 2006), but some source estimates report to be lateralized to the right temporal lobe (Draganova et al., 2008) and others to the left temporal lobe (Pratt et al., 2009). Besides, there is also controversial findings from studies that claim to benefit from TBB due to an enhancement of specific brain wave oscillatory activity, a phenomenon called “frequency following response” or brain wave entrainment (López-Caballero and Escera, 2017). For instance, there was no significant finding between white noise and binaural beat stimulation compared to a white noise control period in an approach to examine the induction of vigilance or cortical states in theta and beta frequency bands from binaural beat stimuli (Goodin et al., 2012). In contrast, binaural-beat stimulation with “beta” frequencies has been shown to increase performance in tasks related to verbal span, working memory, executive functions (Kennerly, 2009) and vigilance (Lane et al., 1998). Moreover, stimulation with binaural beats with frequencies in the range of the EEG gamma band affects divergent thinking (Reedijk et al., 2013) and attentional control (Reedijk et al., 2015). A study with tinnitus patients proved subjectively the effectiveness of BRB and TRT with a decrease in the degree of tinnitus disturbance pre-treatment and post-treatment (David et al., 2010). In this sense, BRB may be used as a tool beyond entraining brain rhythms by focusing on inducing parasympathetic activity (Palaniappan et al., 2015) in tinnitus sufferers that could decrease anxiety (McConnell et al., 2014) and sleep disorders (Chaieb et al., 2015). The increased latency in the ERP components of tinnitus patients to the TBB may be a product of central processes that begin at the brainstem level and adaptive processes that occur upstream cortically, and may represent an enhanced perception to the TBB sound as a learning mechanism that may initially suppress the tinnitus perception (Kaltenbach, 2011). However, results in this study were not significantly different (Table 5) between initial and final sessions; hence, an improvement can be suggested but cannot be proven yet.

### 4.4. Effect of demographic and audiological factors, and AERPs on acoustic therapies

Previous analysis on aging trends in AERPs has revealed that AERP components undergo a gradual quantitative change during aging in the adult life, which contrasts the considerable morphological change that AERPs have before adulthood (i.e., during development) (Kerr et al., 2010). The findings vary across AERP components, but the most agreed upon and reported effect of aging might be the P3 component, which increases latency after 20 years estimates of the rate vary from 0.8−3 ms/year and decreases amplitude (Anderer et al., 1996). The effect of age in P2 and N2, seems to be more controversial (Tomé et al., 2015). In a common consensus, N2 latency increases by about 0.5−1.4 ms/year during aging (Bahramali, 1999). Furthermore, age caused N1 to increase amplitude and latency, but the latter only in posterior brain regions (Anderer et al., 1996). These changes also occur topographically, meaning that AERP components (N1, P2, N2, and P3) change distribution with age. For instance, N1 increases parietally, P2 frontally, N2 and P300 overall in the scalp. No significant differences in age trends were found between female and male groups, but over limited age ranges (e.g., 40−60 years), amplitudes of N2, P2 and P3 had more positive amplitudes in women (Kerr et al., 2010). The shorter latencies that have been reported for women as compared to men have been explained due to brain size and reaction times due to peripheral sex differences, such as muscle mass (Nicholson and Kimura, 1996).

On this evidence, a correlation analysis was run and presented in Figure 14. As a result, the following six variables were significantly correlated (although their correlation was not larger than 50%, in any case): (1) tinnitus intensity, (2) hearing loss, (3) heart rate, (4) acoustic therapy, (5) AUC, and (6) age. Tinnitus has been identified as a multifactorial condition highly associated with hearing loss, age, sex, marital status, education, and even, employment (Brüggemann et al., 2016). However, no conclusive evidence has been found yet. In this study, a significant (but low) correlation was found between tinnitus intensity and right ear hearing loss, left ear hearing loss, heart rate, AUC, and acoustic therapy. Firstly, previous studies (Pinto et al., 2010; Noroozian et al., 2017; Mores et al., 2019) reported no significant correlation between hearing loss and tinnitus severity. Therefore, further investigation is required since the correlation between hearing loss and tinnitus intensity found in this study is low. Secondly, patients with highly distressing tinnitus can eventually have cardiovascular disorders (Brüggemann et al., 2016), and thus, the significant correlation between tinnitus intensity and heart rate found in this study has sense. Thirdly, the significant correlation between tinnitus intensity and acoustic therapy has been demonstrated in the findings discussed in this work. Finally, the significant correlation between tinnitus intensity and AUC demonstrates that this EEG attribute is feasible to evaluate the acoustic therapy effect, in terms of tinnitus intensity.

### 4.5. Implications, limitations, and future work

The current study found that TRT and ADT produced significant neurophysiological changes. On one hand, TRT increased the synchronic neural activity over the right somatosensory region. This increase implies that tinnitus perception augmented, instead of diminished. In line with the THI outcome, 31% of participants in TRT group reported that their tinnitus perception increased after the sound treatment. On the other hand, ADT increased the synchronic neural activity over the occipital region. In contrast to TRT effect, ADT redirected attention, and thus, reduced the tinnitus perception. By way of support, 31% of patients in ADT group reported tinnitus perception. Moreover, ADT was the therapy that presented the lowest side effects, in comparison to the rest of therapies (Figure 7). The combination of these findings provides some support for the conceptual premise that AERPs are useful neuro-patterns to evaluate the neurophysiological effect of acoustic therapies. That is, we are moving toward applying acoustic therapies for tinnitus based on an objective marker, regardless of the patient opinion, which could be affected by his/her own psychological state.

What limits the study is still the auditory stimulus in use. As was mentioned above, the ideal auditory stimulus to evoke a brain response to evaluate the sound effect, or any other treatment, is the tinnitus sound. Unfortunately, the acoustic features of tinnitus are still unknown. In this respect, there is abundant room for further progress in estimating the frequency, intensity, and laterality of tinnitus by means of objective measurements such as AERPs. Once tinnitus had been characterized, a method to monitor tinnitus treatments might be implemented without suppositions and/or adjustments.

## 5. Conclusion

In the present investigation, a method to evaluate the sound effect of acoustic therapies based on the examination of AERPs has been proposed. The findings of the investigation showed that after an 8 week treatment, TRT and ADT, respectively, achieved significant neurophysiological changes over somatosensory and occipital regions. On one hand, TRT increased the tinnitus perception. On the other hand, ADT redirected the tinnitus attention, what in turn diminished the tinnitus perception. THI outcomes verified these neurophysiological findings, revealing that 31% of patients in each group reported that TRT increased tinnitus perception, but ADT diminished it. This study raises the possibility to assign acoustic therapies by neurophysiological response of patient.

## Data availability statement

The datasets presented in this study can be found in online repositories. The names of the repository/repositories and accession number(s) can be found below: https://data.mendeley.com/datasets/kj443jc4yc/1.

## Ethics statement

The clinical protocol was approved by the Ethical Committee of the Medicine School at Tecnologico de Monterrey (COFEPRIS13CI19039138) and registered as clinical trial in BioMed Central (ISRCTN14553550). The database is publicly available at https://data.mendeley.com/datasets/kj443jc4yc/1. The patients/participants provided their written informed consent to participate in this study.

## Author contributions

LA-V and DI-Z designed and implemented the study protocol, undertook the experimental procedure, proposed the signal analysis strategy, undertook the signal pre-processing and processing, and wrote the manuscript draft and final version. AT-T programmed and applied the machine learning algorithms, generated all the figures concerning method section, and assisted in signal processing. DZ contextualized the framework, interpreted the results and neurologically speaking. NN-R assisted in signal processing, generated all the figures, and wrote the Introduction section along with DZ. JA-G pre-processed raw data and extracted the AERPs of most of the files. All authors contributed to the article and approved the submitted version.
